# A fine-grained image classification algorithm based on self-supervised learning and multi-feature fusion of blood cells

**DOI:** 10.1038/s41598-024-74753-2

**Published:** 2024-10-03

**Authors:** Nan Jia, Jingxia Guo, Yan Li, Siyuan Tang, Li Xu, Liang Liu, Junfeng Xing

**Affiliations:** 1 Baotou Medical College, Baotou, 014040 Inner Mongolia China; 2 Baotou Central Hospital, Baotou, 014040 Inner Mongolia China

**Keywords:** Leukaemia, Computational science

## Abstract

Leukemia is a prevalent and widespread blood disease, and its early diagnosis is crucial for effective patient treatment. Diagnosing leukemia types heavily relies on pathologists’ morphological examination of blood cell images. However, this process is tedious and time-consuming, and the diagnostic results are subjective, leading to potential misdiagnosis and underdiagnosis. This paper proposes a blood cell image classification method that combines MAE with an enhanced Vision Transformer to tackle these challenges. Initially, pre-training occurs on two datasets, TMAMD and Red4, using the MAE self-supervised learning algorithm. Subsequently, the pre-training weights are transferred to our improved model.This paper introduces feature fusion of the outputs from each layer of the Transformer encoder to maximize the utilization of features extracted from lower layers, such as color, contour, and texture of blood cells, along with deeper semantic features. Furthermore, the dynamic margins for the subcenter Arcface Loss function are employed to enhance the model’s fine-grained feature representation by achieving inter-class dispersion and intra-class aggregation. Models trained using our method achieved state-of-the-art results on both the TMAMD dataset and Red4 dataset, with classification accuracies of 93.51% and 81.41%, respectively. This achievement is expected to be a valuable reference for physicians in their clinical diagnoses.

## Introduction

Leukemia, a common malignant disease of the hematological system, is typically categorized into myeloid and lymphoid lineages^1^. Depending on the degree of cell differentiation and the course of the disease, it is further classified as acute or chronic. Therefore, the four main types of leukemia observed in clinical practice are acute myeloid leukemia (AML), chronic myeloid leukemia (CML), acute lymphoblastic leukemia (ALL), and chronic lymphocytic leukemia (CLL)^2^. Cytomorphology serves as a vital method for diagnosing and assessing hematological disorders. Identifying leukocyte types in bone marrow or peripheral blood, along with determining their numbers or percentages, aids in diagnosing the presence and type of disease^3^^4^,.However, this task demands specialized medical knowledge and extensive practical experience. Moreover, it is time-consuming and fatiguing, leading to potential misdiagnoses or omissions.Thus, developing a rapid, accurate, and objective method for diagnosing leukemia cell morphology holds significant importance for clinical diagnosis, pathological analysis, and treatment selection. Additionally, intelligent recognition of leukemia cells plays a crucial role in medical education and other areas.

## Related work

With the continuous advancement of artificial intelligence in medical imaging, researchers worldwide have conducted extensive studies on the classification and recognition of leukocytes in bone marrow smears. These efforts primarily fall into two main categories: traditional machine learning and deep learning.In machine learning methods, the process can be summarized in three steps: Accurate segmentation of leukocyte nuclei and cytoplasm. This involves commonly used segmentation methods such as threshold-based segmentation, edge detection-based segmentation, and clustering-based segmentation. These algorithms primarily rely on factors like pixel intensity, neighboring pixel differences, and changes in texture features within clinical cell images. Manual extraction of image features from the segmented regions, including color, gradient, and texture. Common methods for this step include gradient histogram analysis, scale-invariant feature transformation, local binary mode, and local directional mode. Screening the extracted features and inputting them into a classifier to complete the classification of leukocytes.These methods, extensively researched both domestically and internationally, contribute significantly to the development of automated leukocyte classification techniques^5-13^.

Deep learning possesses the ability to automatically extract features due to its powerful learning capabilities and high adaptability. Convolutional Neural Networks (CNNs) and Vision Transformers are extensively employed for various tasks such as classification^14-24^, semantic segmentation^25-28^, and detection^29-32^of blood cell images.For single cell classification, Matek et al^21^. constructed the largest publicly available dataset of microscopic images of individual bone marrow cells, comprising over 170,000 images of leukocytes across 21 categories. They utilized the ResNext model for classification, achieving over 90% accuracy for neutrophils, promyelocytes, lymphocytes, and erythrocytes.Tripathi et al^22^. utilized the same dataset, selecting 17 categories and training it using the CoAtNet model, a hybrid model merging CNN and Transformer architectures. They achieved over 90% accuracy across all 10 categories.Tianyu Sun et al^23^. proposed a blood cell image recognition method based on an enhanced Vision Transformer, incorporating a sparse attention module to improve the model’s ability to express fine-grained features and a contrast loss function. Their experimental results showed an accuracy rate of 91.96% on the Munich blood cell morphology dataset.Ye Wang et al^24^. developed a large-scale fine-grained erythrocyte image dataset with four classification categories, containing 5666 individual erythrocyte images. They designed a novel shape-aware image classification network for classification, achieving an 81.12% accuracy rate.

Although the aforementioned studies have made significant strides in blood cell classification, they exhibit certain limitations. Some studies focus solely on major blood cell classes, neglecting the ability to distinguish finer subclasses. For instance, granulocytes can be categorized into primitive, early juvenile, intermediate juvenile, and late juvenile stages based on their growth stage. While some studies address the classification of cell subclasses, there remains room for improving their classification accuracy.In this paper, we propose a self-supervised learning method based on mask image modeling for blood cell classification. Initially, the MAE model undergoes self-supervised learning on a larger dataset, followed by fine-tuning its backbone model for the target dataset’s classification task. Additionally, to enhance the model’s ability to discriminate between subclasses, techniques such as fusing the outputs of each encoder layer and employing dynamic margins for subcenterArcFace Loss are utilized to improve fine-grained classification accuracy.

The primary contributions of this study are outlined as follows:

1. Introducing a self-supervised learning approach for MAE represents the first application of this technique to blood cell classification. Initially, the MAE encoder undergoes pre-training by merging two public datasets to form the training set for MAE. Subsequently, the pre-training weights of the encoder are transferred to the blood cell classification model. It is observed that the training weights derived from MAE significantly outperform the pre-training weights obtained from ImageNet1K for the blood cell classification task.
2. The dataset utilized in this paper comprises images with subtle differences between various categories, making it challenging for doctors to differentiate between certain categories accurately. To achieve fine-grained classification of these images, the dynamic margins for the subcenterArcFace Loss function, an enhancement of Arcface, have been implemented. This approach facilitates improved convergence of intra-class distances and separation of inter-class distances in the angular domain, thereby enhancing the accuracy of blood cell classification.
3. We propose a combined learning scheme that combines the above contributions. Experiments are conducted on 2 different blood cell classification datasets, and the results show that the proposed method outperforms existing methods.

### Dataset and preprocessing

#### Dataset

In this paper, two publicly available datasets were utilized. The first dataset is a peripheral blood smear dataset established by Matek et al^23^.. It comprises 18,365 expert-labeled single-cell images spanning 15 categories. These images were collected from peripheral blood smears of 100 patients diagnosed with acute myeloid leukemia and 100 patients without signs of hematological malignancy at the University Hospital Munich from 2014 to 2017. However, this dataset contains multiple cells in some images. To address this, Chinese scholars Sun Tianyu et al^24^. processed the dataset by employing a combination of manual and automated methods to crop the target cells in each image based on their longest diameter. Due to imbalanced categories in the dataset, categories with fewer than 30 images were removed, retaining only those with more than 30 images. Additionally, categories with fewer data points were augmented through flipping and rotating, while those with more data were randomly undersampled. The data distribution of the processed dataset is presented in Table 1.

**Table 1:** Distribution of data in the TMAMD dataset.

Cell class	Training Set	Validation Set	Quantity
NGS	800	200	1000
NGB	435	110	545
LYT	800	200	1000
MON	800	200	1000
EOS	678	170	848
BAS	315	80	395
MYO	800	200	1000
PMO	280	70	350
MYB	165	45	210
EBO	310	80	390
Total	5383	1355	6738

Myeloblast(MYO), Promyelocyte(PMO), Myelocyte(MYB), neutrophil stab granulocyte (NGB), neutrophil segmented granulocyte (NGS), Basophil(BAS), Eosinophil(EOS), Lymphocyte(LYT), Erythroblast(EBO), Monocyte(MON).

**Fig. 1:**
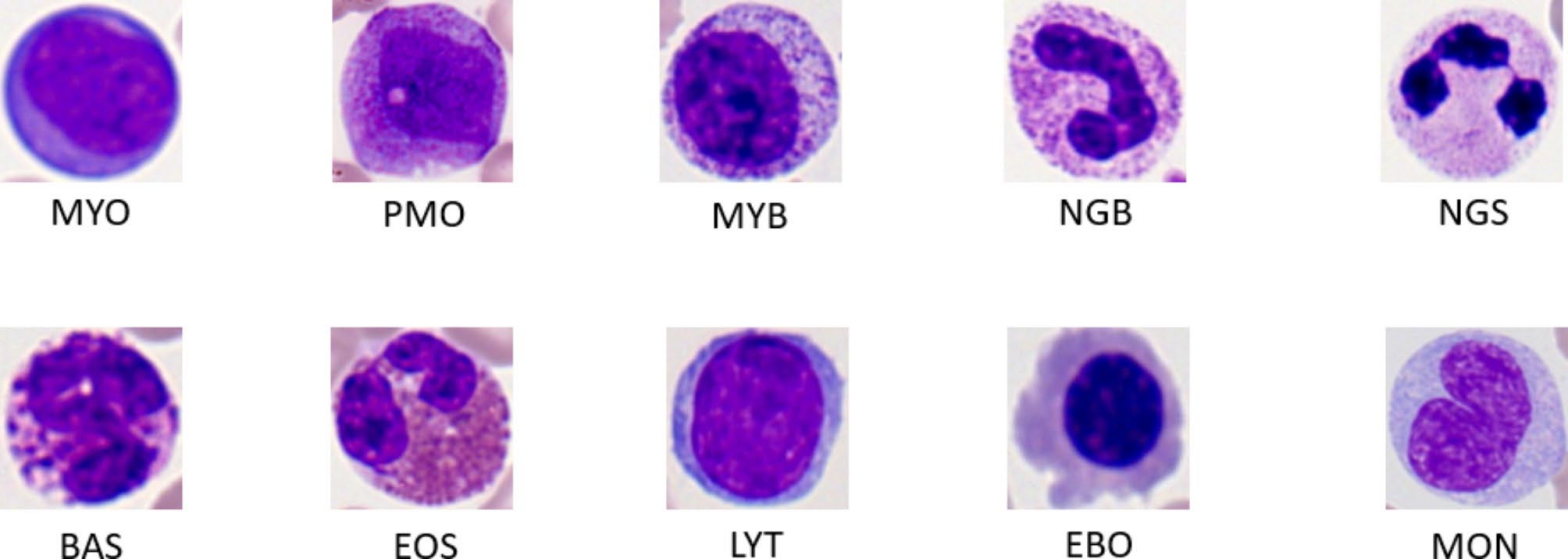
Example of TMAMD cell image.

Sample images of each cell type are shown in Fig. 1, and the data were downloaded from https://github.com/sty16/cell_transformer.

The second dataset, known as the red4 dataset^25^, was compiled using data from the Second Hospital of Jilin University in Changchun, China. It comprises images of four types of erythroid cell subtypes annotated by three hematologists, totaling 5,666 images. The dataset distribution is presented in Table 2.

**Table 2:** Distribution of data in the red4 dataset.

Dataset	Pro	Bas	Pol	Ort	Quantity
Training Set	146	428	1702	1589	3865
Validation Set	28	69	312	246	655
Test Set	48	115	526	457	1146
Total	222	612	2540	2292	5666

proerythroblast cells (Pro), basophilic erythroblast cells (Bas), polychromatophilic erythroblast cells (Pol), orthochromic erythrobla cells (Ort).

**Fig. 2:**
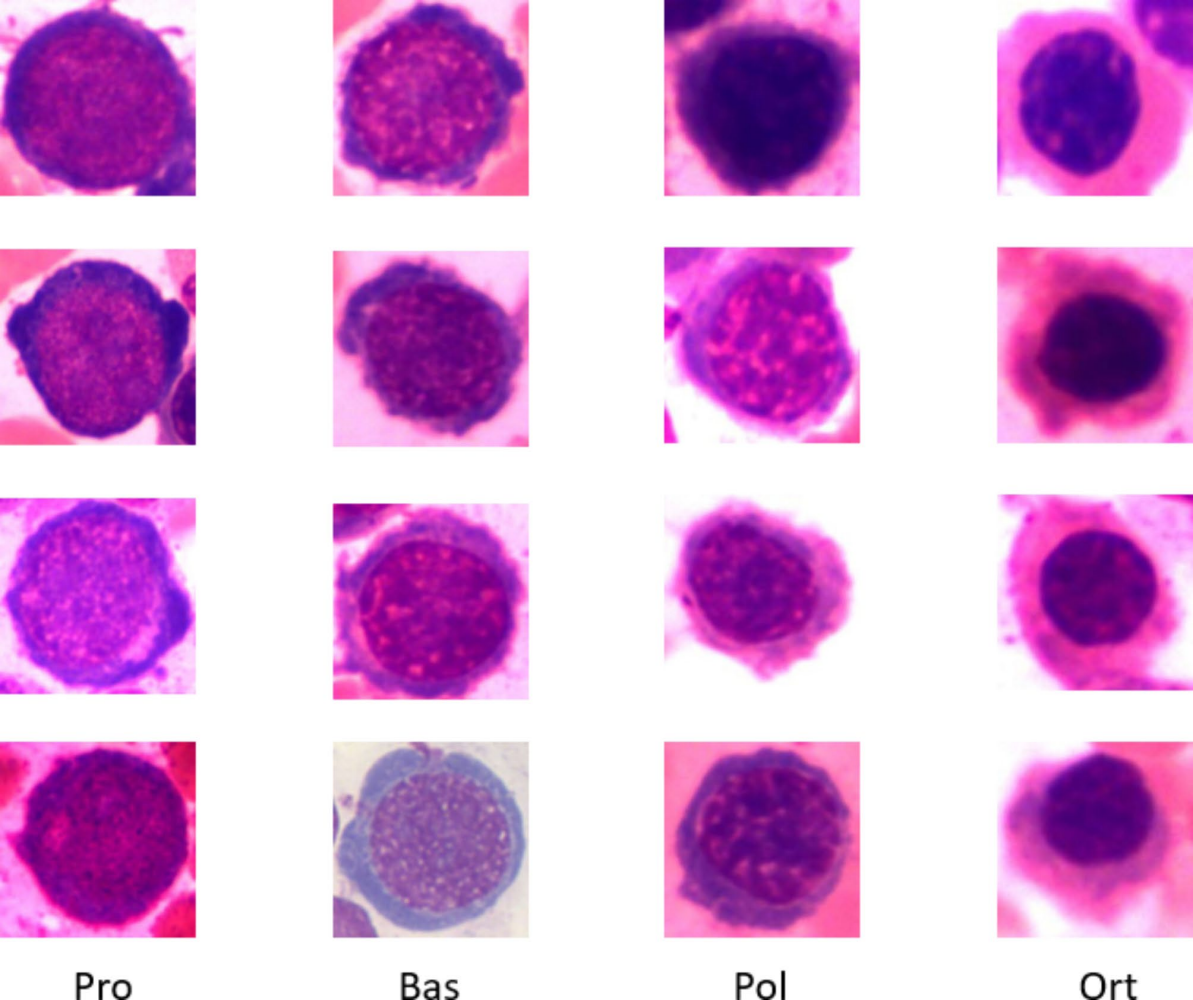
Example of red4 cell image.

Sample images of each cell type are shown in Fig. 2 and the data were downloaded from https://github.com/wangye8899/BMEC.

## Preprocessing

Due to the disparity in image sizes between dataset 1 and dataset 2, a preprocessing technique is employed. Initially, the image is resized to 224 × 224 using the double cubic interpolation algorithm. Subsequently, random horizontal flipping, random enhancement^33^, and random erasure^34^ are applied. Random enhancement encompasses various data enhancement strategies, such as automatic contrast, histogram equalization, rotation, flipping, exposure adjustment, color balance, contrast adjustment, brightness adjustment, and sharpening. For this purpose, two enhancement methods are randomly selected from the pool of available methods each time. The total number of enhancement levels is set to 10, with each enhancement having 9 levels. The probability of random erasure is set to 0.25, with the minimum erased area ratio set to 0.02 and the maximum erased area ratio set to 0.33.

## Methods

**Fig. 3:**
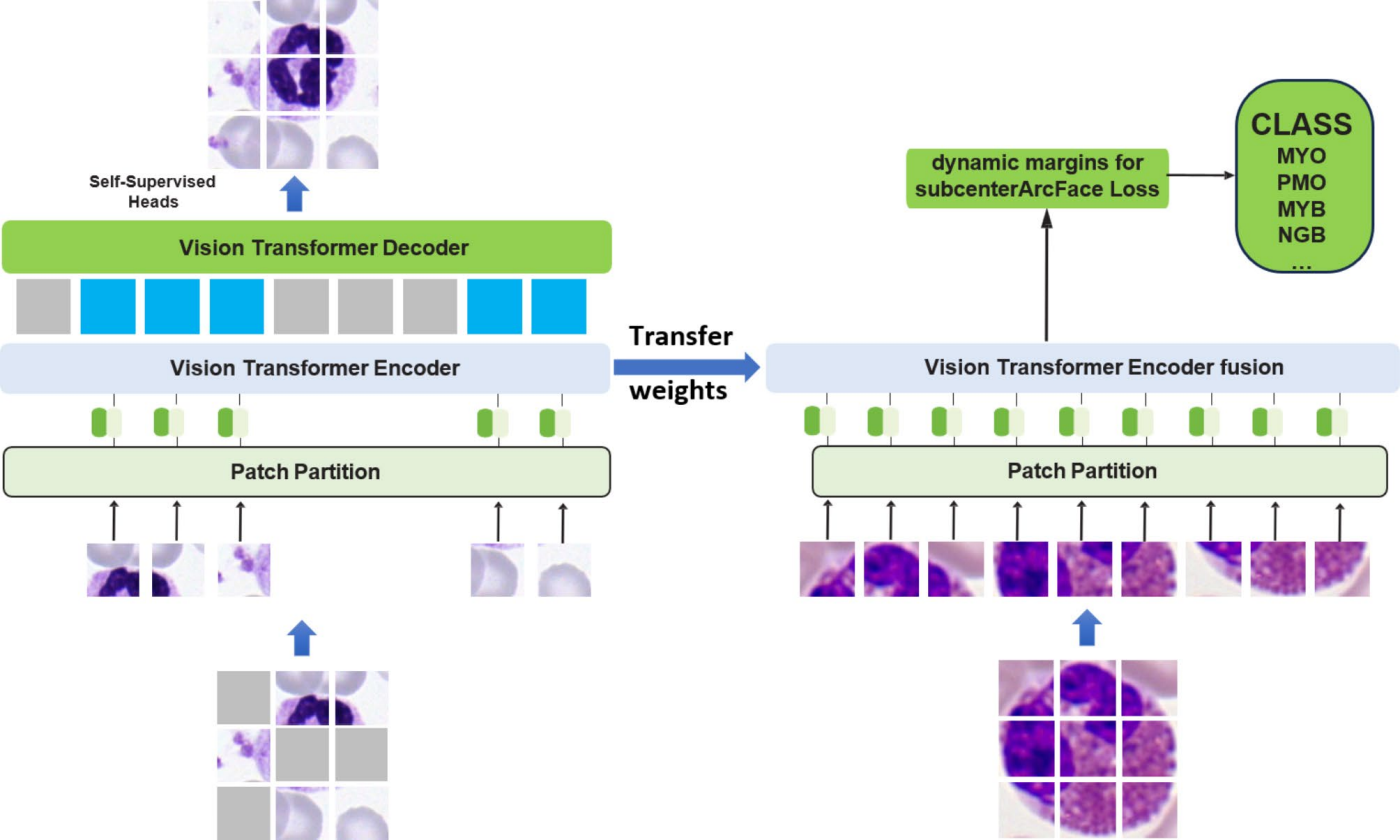
Blood cell classification network pipeline.

Our pipeline, depicted in Fig. 3, involves several steps. Firstly, dataset 1 and dataset 2 are merged into a single dataset. Next, the merged dataset undergoes self-supervised learning using MAE (Masked Auto-encoders)^35^ to obtain a pre-trained model. Subsequently, the weights of this pre-trained model are transferred to an improved Transformer model. This enhanced model incorporates feature fusion of the outputs of each layer of the encoded code and utilizes the dynamic margins for the subcenterArcFace Loss function. Finally, the model is trained separately on dataset 1 and dataset 2.

## Vison transformer(VIT)

**Fig. 4:**
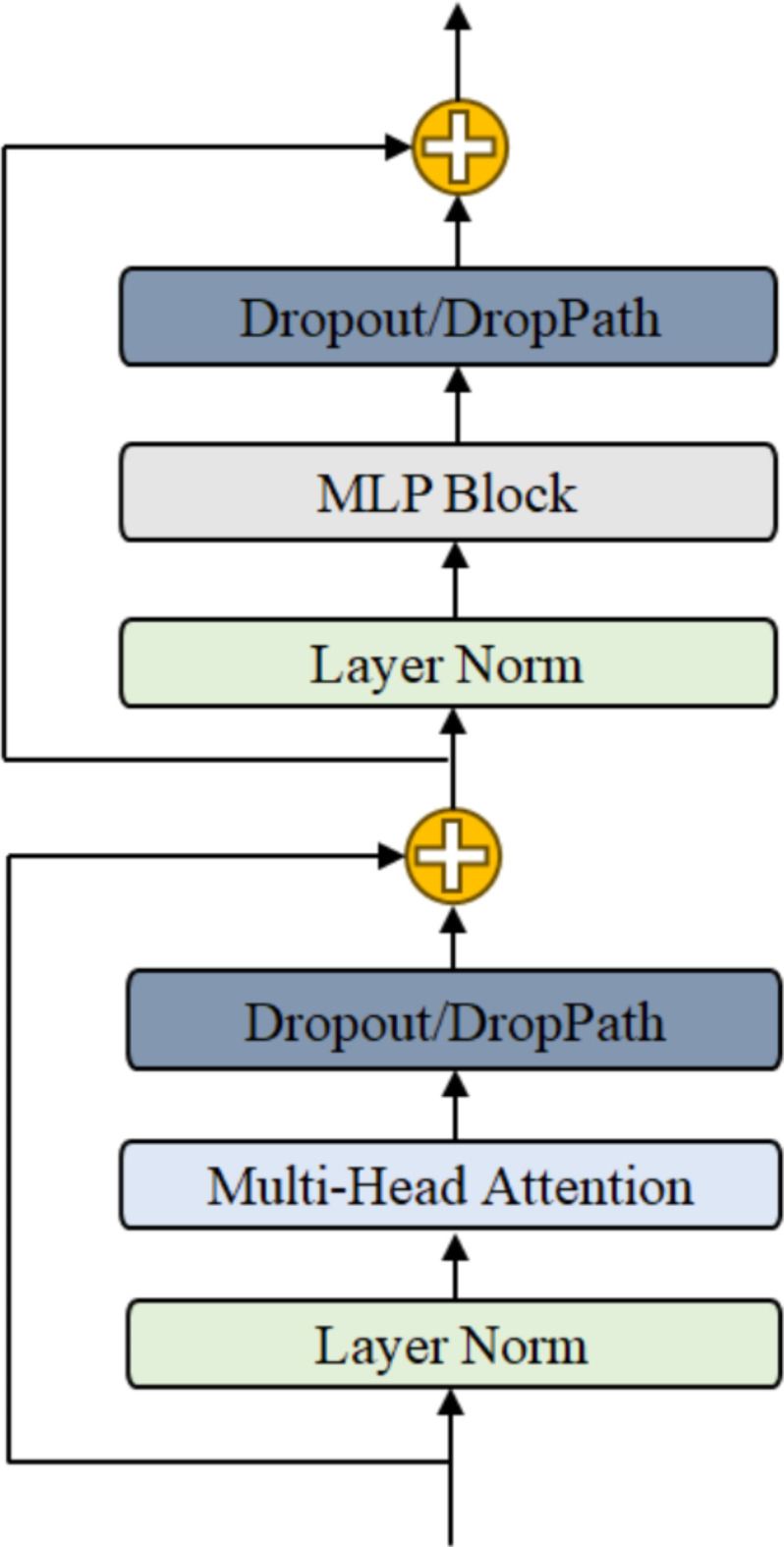
VIT Encoder Block.

In this paper, the VIT (Vision Transformer) model^36^ is utilized for MAE (Masked Auto-encoders) self-supervised learning, and the classification network for blood cells is enhanced based on the traditional VIT model. The traditional VIT comprises two main components: Image division embedding: The VIT model requires input data to be serialized, necessitating the division of the image into multiple smaller pieces to form a vector sequence input for the model. This can be accomplished directly through a convolutional layer in code implementation. For classification tasks, an additional classification vector is required as the output vector of the final features for image classification. Since the embedded vectors lack positional information, positional encoding is superimposed onto each vector. In this experiment, sine-cosine encoding is utilized for positional encoding. Encoder: VIT’s encoder consists of L coding modules, with each module comprising a multi-head self-attention module (MSA) and a multi-layer perceptron (MLP), among other components. The structure of the encoder is illustrated in Fig. 4.

### Masked autoencoders model (MAE)

**Fig. 5:**
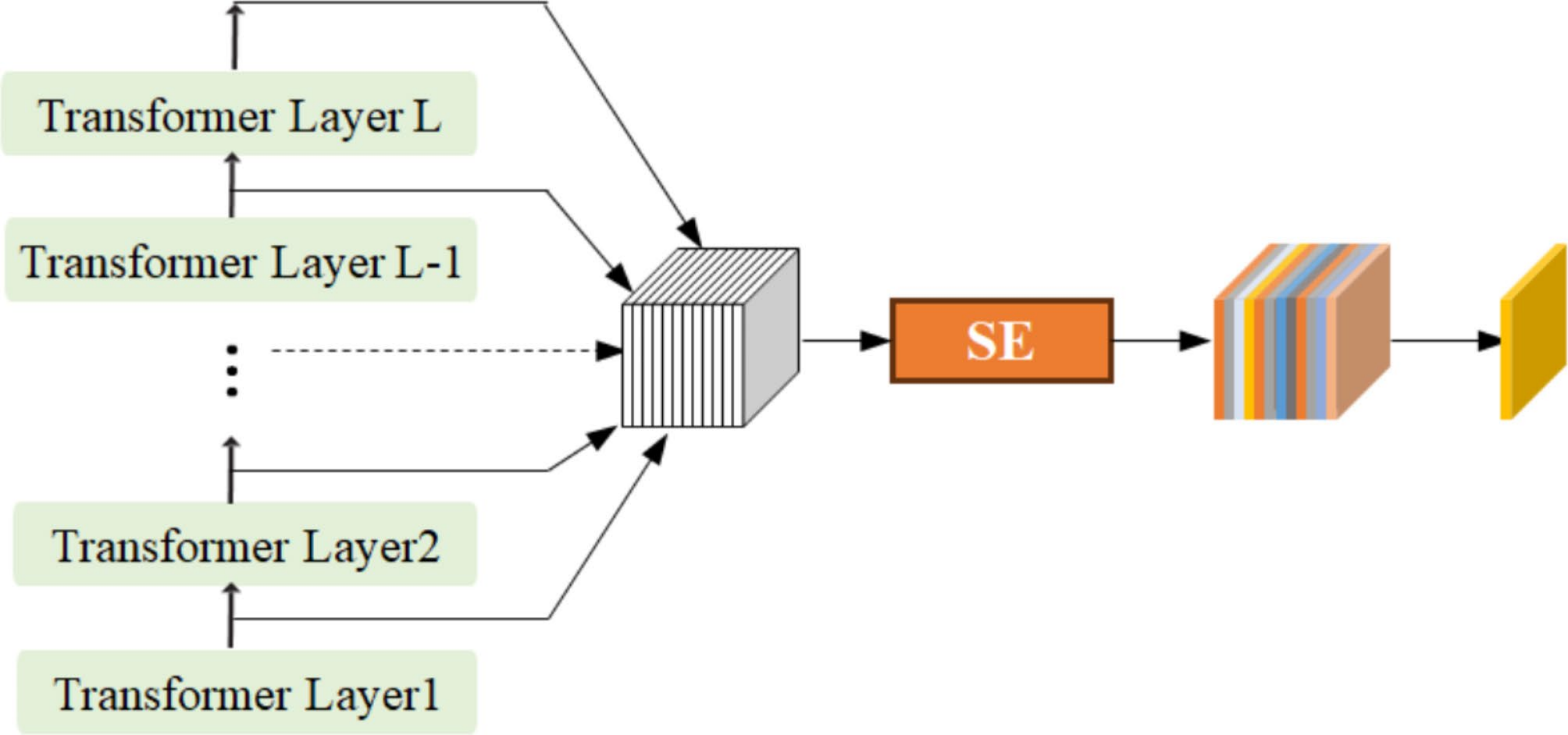
Structure of Vison Transformer Encoder fusion.

The structure of MAE is depicted in the left half of Fig. 5. Initially, an image is divided into several small blocks. Subsequently, a portion of these image blocks is randomly masked, with the masking proportion set to 75% in this paper. The model is then trained on a large dataset to comprehend the semantic information of the image and reconstruct the masked-off image blocks. MAE consists of three parts: image division embedding, encoder, and decoder. The first two parts are akin to those in the VIT model, where only the unmasked image blocks serve as input. The decoder, on the other hand, requires both the masked vectors and the encoder output vectors as inputs, along with positional encoding. Each element in the decoder output represents a small block of pixel value vectors. The final layer of the decoder is a linear mapping with the number of output channels equal to the number of pixel values in the chunk, resulting in the image’s reconstruction. It’s worth noting that the decoder solely serves as an auxiliary tool module for pre-training and is not utilized for downstream tasks.

## Vison transformer encoder fusion

In the blood cell classification task, the feature extraction network utilized is the Vision Transformer Encoder fusion, which is an enhanced model derived from the VIT-base. The structure of this module is illustrated in Fig. 5. Notably, the cls_token in the VIT encoder is eliminated. Instead, the output of each layer of the VIT encoder undergoes a splicing operation. Subsequently, it is passed through the SE (Squeeze-and-Excitation) module^37^. The output of the SE module is then summed up along the dimension of dim = 1 to form the final features of the model.

**Algorithm 1:**
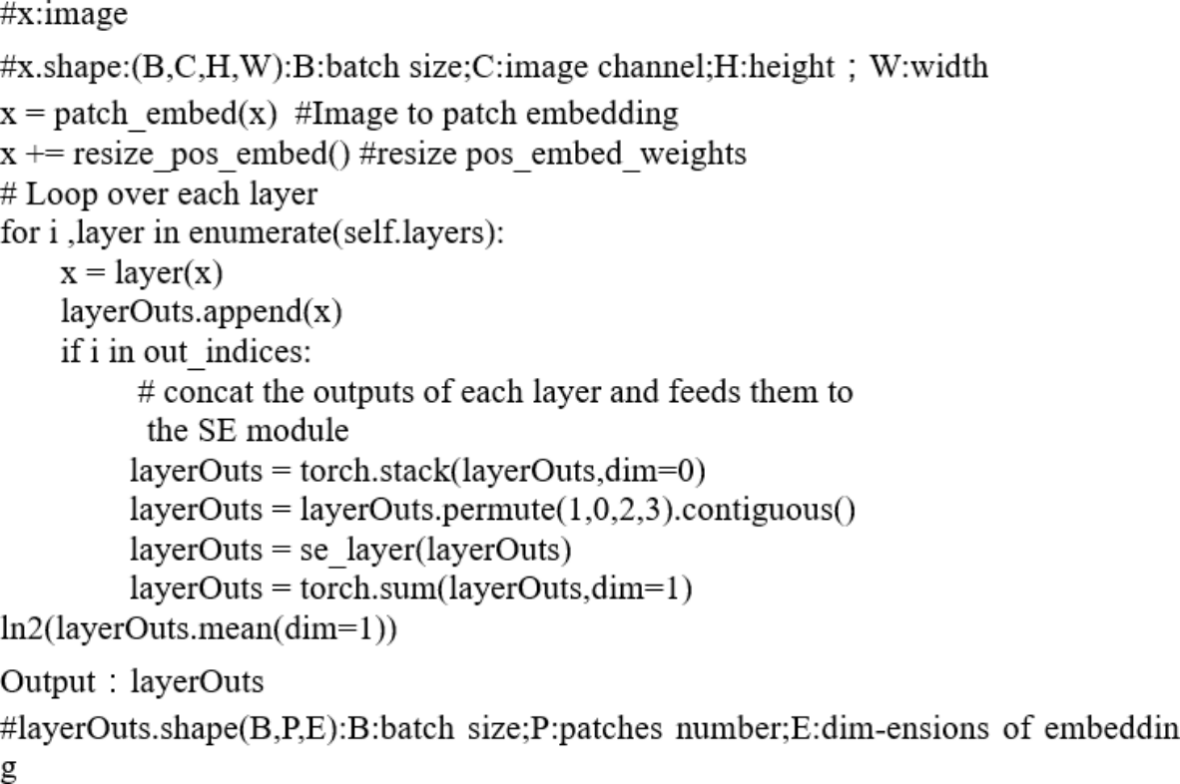
The Pseudo-code of Vison Transformer Encoder fusion on pytorch

## Dynamic margins for subcenterarcface loss

The traditional softmax Cross-Entropy (CE) Loss solely promotes feature separability. In our approach, we utilize the dynamic margins for subcenter-Arcface Loss^38^. ArcFace^39^is a loss function based on angular cosine, which introduces angular spacing concepts in the feature space. Its objective is to cluster sample features of the same category while ensuring sufficient separation between features of different categories.Subcenter^40^ enhances ArcFace by introducing subclasses, thereby relaxing intra-class constraints to improve global feature quality and classification accuracy. By introducing subclasses, we alleviate the constraint that forces all samples to be close to the corresponding orthocenter for each class. For our implementation, we choose the number of sub-centers per class, denoted as K, to be 3.

Our training dataset exhibits class imbalance, so we employ dynamic margins. This approach ensures better model convergence in the presence of imbalanced classes. Smaller classes are assigned larger margins as they are inherently harder to learn.

Through the dynamic margins for subcenter-Arcface Loss, our experimental results demonstrate the attainment of highly discriminative features for robust cell classification. The formula for the dynamic margins for subcenter-Arcface Loss is as follows:

1 $$L= - \log \frac{{{e^{s\cos ({\theta _{i,{y_i}}}+m)}}}}{{{e^{s\cos ({\theta _{i,{y_i}}}+m)}}+\sum\nolimits_{{j=1,j \ne {y_i}}}^{N} {{e^{s\cos {\theta _{i,j}}}}} }}$$,

where ${\theta _{i,j}}=ar\cos \left( {{{\hbox{max} }_k}\left( {W_{{jk}}^{T}{X_i}} \right)} \right),k \in \left\{ {1, \cdot \cdot \cdot ,K} \right\}$,$m=(a - b) \cdot {n^{ - \lambda }}+b$, s = 64 is the feature re-scale parameter, and N is the total class number. K is the number of subcenters, in this experiment we take K = 3, n is the number of pictures in each category, a, b and λ are parameters, λ = -0.5 in the experiment, a and b are the upper and lower bounds of the margin function, a = 0.5 and b = 0.05 in the experiment.

### Experimental results and analysis

#### Implement detail

All models in this experiment were trained on an NVIDIA GeForce RTX 3090 graphics card running the Linux operating system. The deep learning framework used for training was PyTorch 2.1.1.

For MAE training, the dataset used is the ensemble of TMAMD and red4. The backbone model employed is vit-base-p16, with mask_ratio = 0.75 and epoch = 1600. The classification model is the improved VIT-base model. The learning rate is determined using the Multiple learning rate schedule, where the first 20 epochs utilize linear warm-up by epoch, followed by a CosineAnnealing schedule. The optimizer used is AdamW, with weight_decay set to 5e-4. The learning rate is initialized to 6.25e-5. The entire training process is halted at the 400th epoch.

### MAE reconstruction

**Fig. 6:**
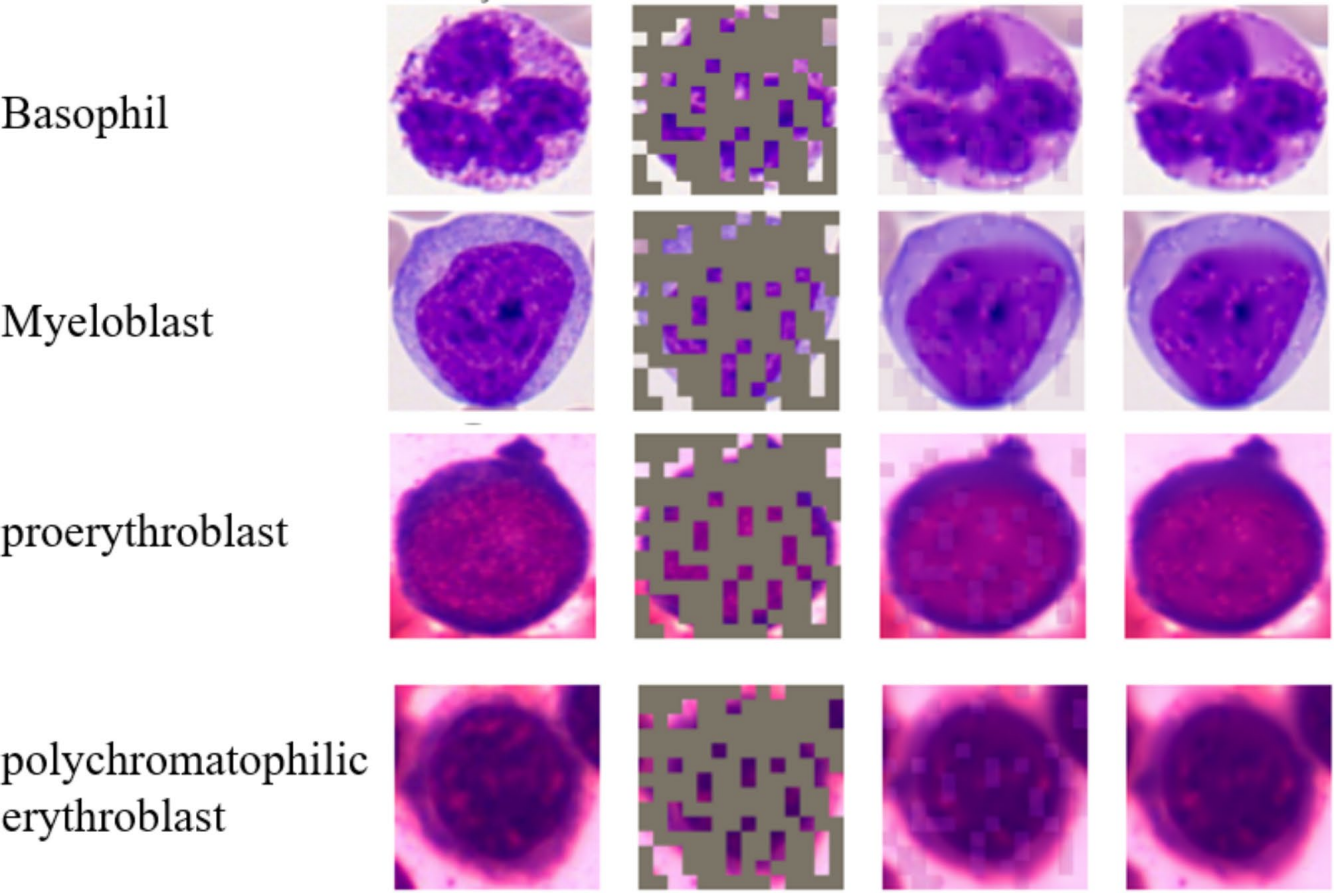
MAE reconstruction results.

We present the reconstruction results for MAE with 75% masking in Fig. 6. The four columns display the original image, masked image, reconstructed image, and reconstruction + visible image, respectively. The results demonstrate that MAE successfully retrieves lost information from the random context. However, since the reconstruction loss is solely applied to the masked patches, the recovered visible patches appear blurrier, akin to observations in natural images. It is important to emphasize that the primary objective of MAE is to enhance our hematocrit classification task rather than generate high-quality reconstructions.

### Classification result

**Fig. 7:**
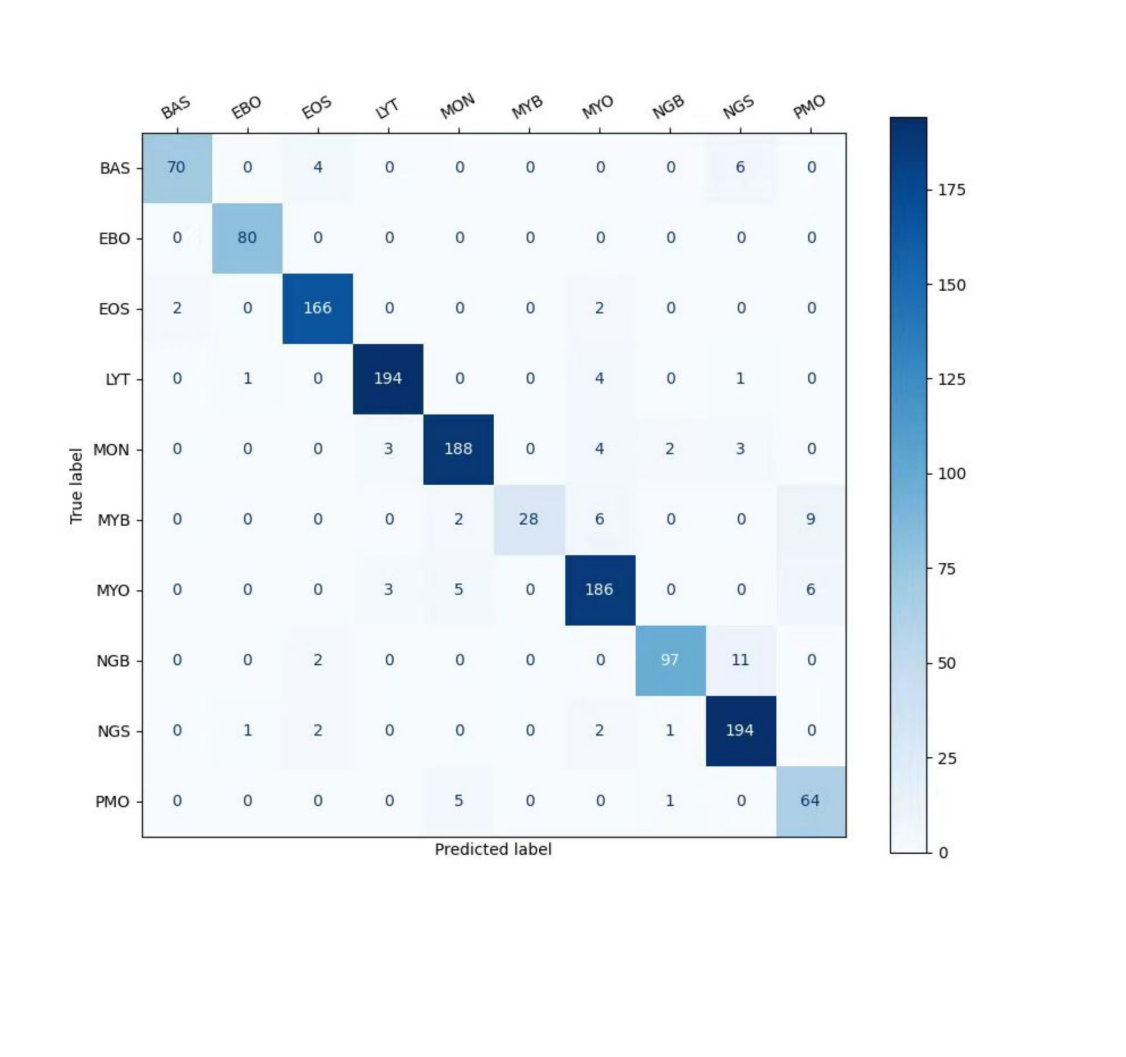
The confusion matrix of the proposed method.

The average classification accuracy of this paper’s method on the TMAMD dataset is 93.51%, and the precision, recall, and F1-score for each category are shown in Table 3, and the confusion matrix is shown in Fig. 7.

**Table 3:** The precision, recall, and F1-score of the proposed method.

Cell_cls	Pre(%)	Rec(%)	F1(%)
BAS	97.22	87.50	92.11
EBO	97.56	100.0	98.77
EOS	95.40	97.65	96.51
LYT	97.00	97.00	97.00
MON	94.00	94.00	94.00
MYB	100.0	62.22	76.71
MYO	91.18	93.00	92.08
NGB	96.04	88.18	91.94
NGS	90.23	97.00	93.49
PMO	81.01	91.43	85.91

The cell prediction results for EBO, EOS, LYT, MON, MYO, and NGS demonstrate strong agreement with the real labels, with Precision, Recall, and F1-score all exceeding 90%.

This paper conducts a comparison with current mainstream deep learning models including ResNet^41^, VGG^42^, EfficientNet^43^, EfficientNetV2^44^, MobileNetV2^45^, MobileNetV3^46^, Vision Transformer, and Swin Transformer^47^. Table 4 presents the comparison results of these different models on the TMAMD dataset.

**Table 4:** The performance of different methods.

Method	Bankbone	Acc(%)	Pre(%)	Rec(%)	F1(%)	Flops	Params
VGG	VGG16	91.66	88.91	87.16	87.78	15.47G	0.134G
Restnet	Restnet101	92.10	90.40	88.22	88.91	7.83G	42.52 M
Restnext	Restnext101	90.33	91.03	85.86	86.97	16.47G	86.76 M
EfficientNet	b3	91.66	89.84	87.40	88.19	0.99G	10.71 M
EfficientNet	b5	92.62	91.09	88.37	89.35	2.41G	28.36 M
EfficientNetV2	b3	91.51	89.29	86.82	87.15	1.63G	12.84 M
MobileNetV2		92.18	91.27	88.00	89.32	0.31G	2.24 M
MobileNetV3	large	89.82	88.92	85.61	86.26	0.22G	4.22 M
Vision Transformer	vit-base-p16	92.10	91.09	89.91	90.39	16.87G	85.81 M
Swin-Transformer	Swin-b	92.62	92.16	88.50	89.25	15.47G	86.75 M
**Ours**	vit-base-p16	93.51	93.96	90.80	91.85	16.78G	85.82 M

**Fig. 8:**
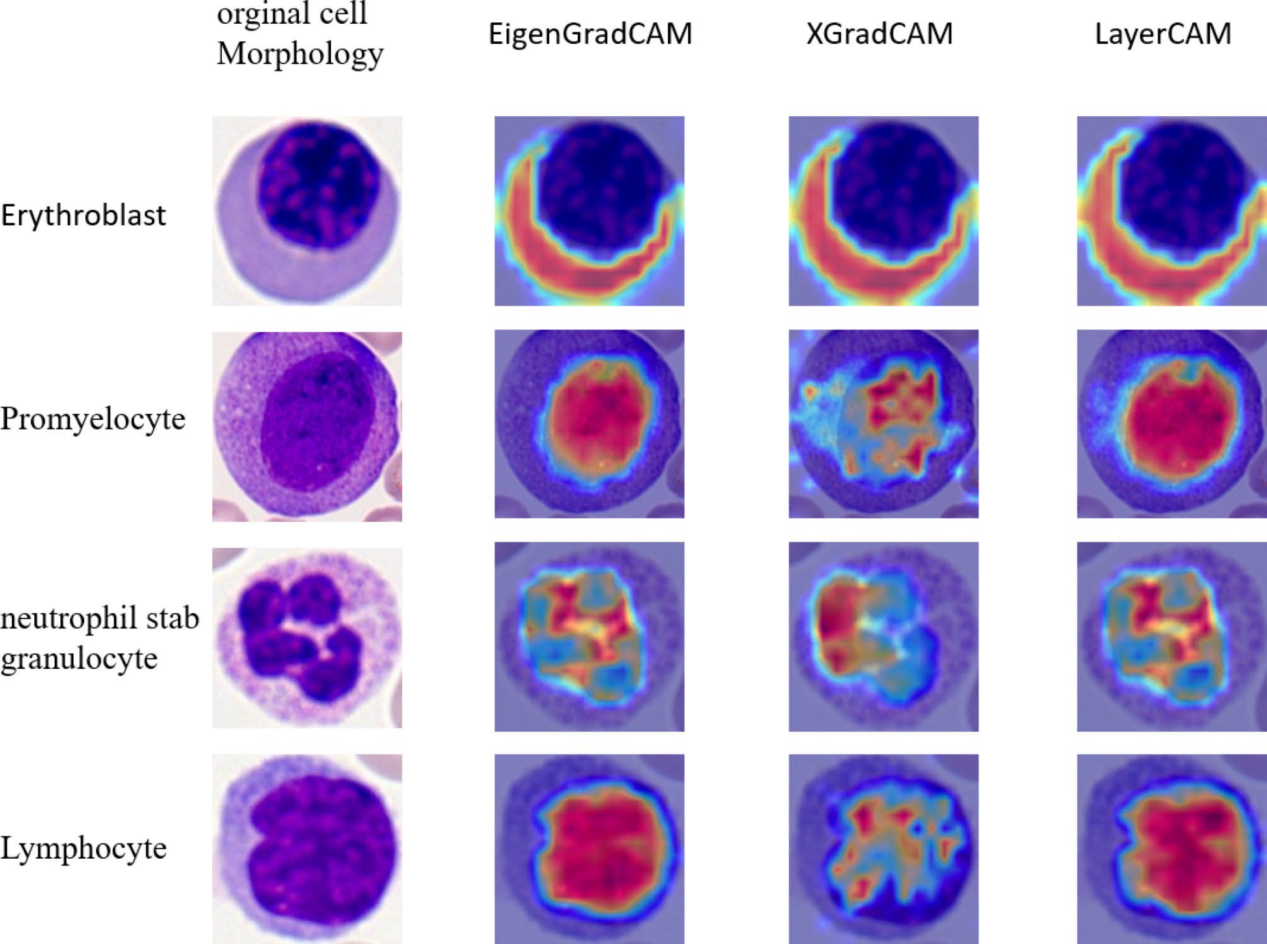
Visualization results of self-attention maps.

The self-attention map within our model is depicted in Fig. 8. We utilized EigenGradCAM, XGradCAM, and EigenGradCAM algorithms for our analysis. Four randomly chosen blood cell images from the dataset were analyzed. The first column in the figure displays results obtained using the EigenGradCAM algorithm, the second column shows results from the XGradCAM algorithm, and the third column presents results from the LayerCAM algorithm. All three algorithms overlay heatmaps on the original images to highlight regions containing crucial information relied upon by the model for image classification, with these key areas emphasized in red. The analysis indicates that the network has been trained to focus exclusively on the cells themselves, disregarding the background of the images. Essentially, the network has learned to concentrate on the cytoplasmic organization or nuclear structure of individual cell images, enabling predictions based on these meaningful features.

Additionally, employing the method outlined in this paper, we also achieved State-of-the-Art performance on the Red4 dataset, with an average classification accuracy of 81.41%.

### Ablation study

We conducted an ablation experimental study of the method outlined in this paper to analyze the effect of different modules on cell classification. We evaluated the impact of the fusion of attention feature outputs of each layer, dynamic margins for subcenterArcFace loss, and different pre-training weights on the classification of cell images, respectively.

**Table 5:** Ablation study on different improvement.

Method	improvement	Acc	Pre	Rec	F1
vit-base-p16	Imagenet1K pretrain	92.10	91.09	89.91	90.39
vit-base-p16	Imagenet1K pretrain + fusion	92.62	91.08	90.24	90.58
vit-base-p16	Imagenet1K pretrain + fusion + dynamic margins for subcenterArcFace	93.21	92.17	90.42	91.10
vit-base-p16	MAEprtrain + fusion + dynamic margins for subcenterArcFace	93.51	93.96	90.80	91.85

The vit-base-p16 model consists of a total of 12 stacked Encoders. The lower encoder layers primarily extract detailed features such as brightness, contour, and edge points. With the deepening of the layers, the model can further extract deeper features. To fully leverage the features of each layer, this paper employs the SE module to fuse the features of each layer, focusing on those encoder layers that are more meaningful for cell classification. The output of the encoder layers is thus more meaningful for cell classification. As demonstrated in Table 5, the classification accuracy is improved by 0.52% through the use of the encoder fusion module.

**Fig. 9:**
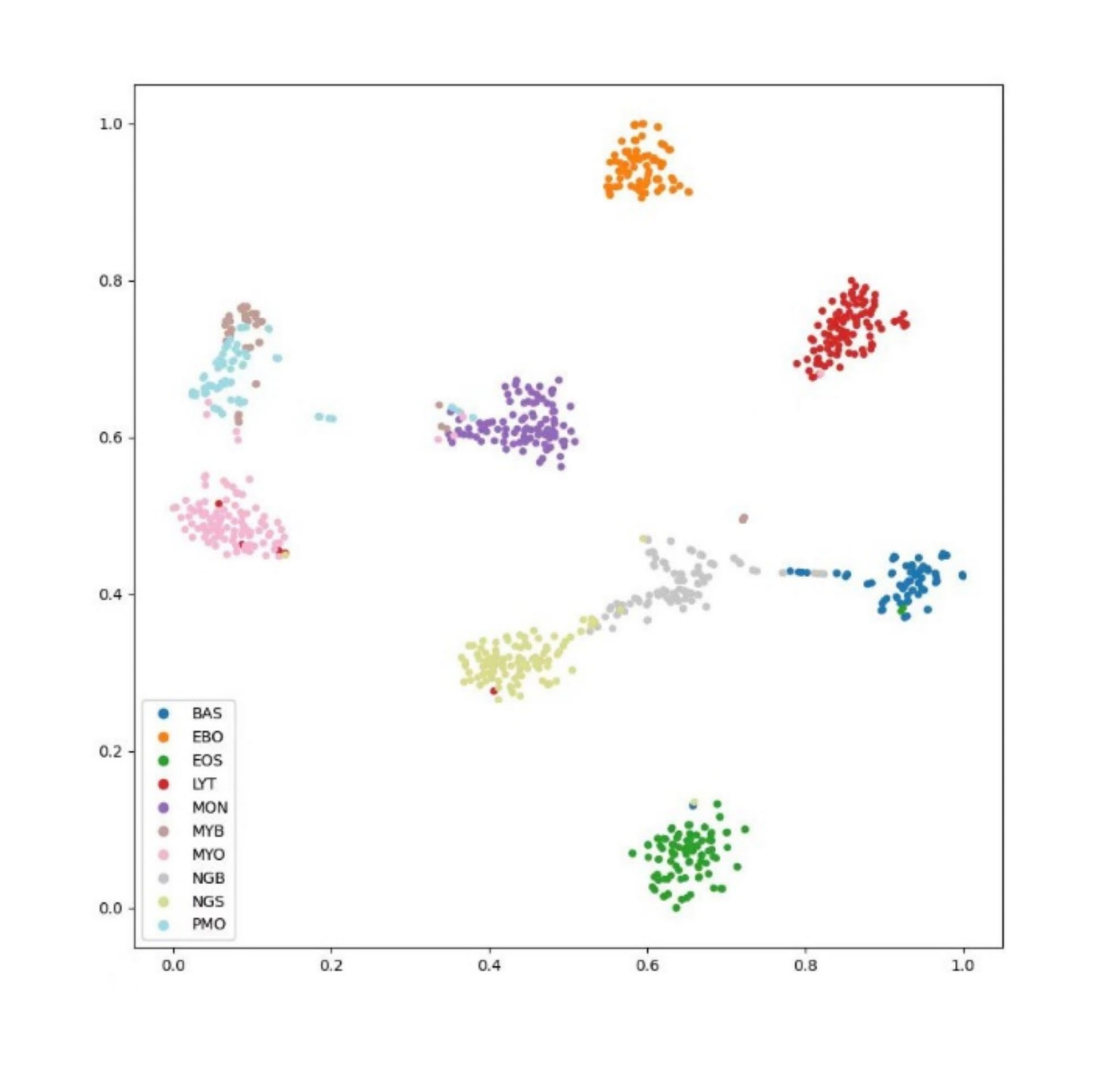
t-SNE dimensional reduction for categorical features with softmax CE Loss.

**Fig. 10:**
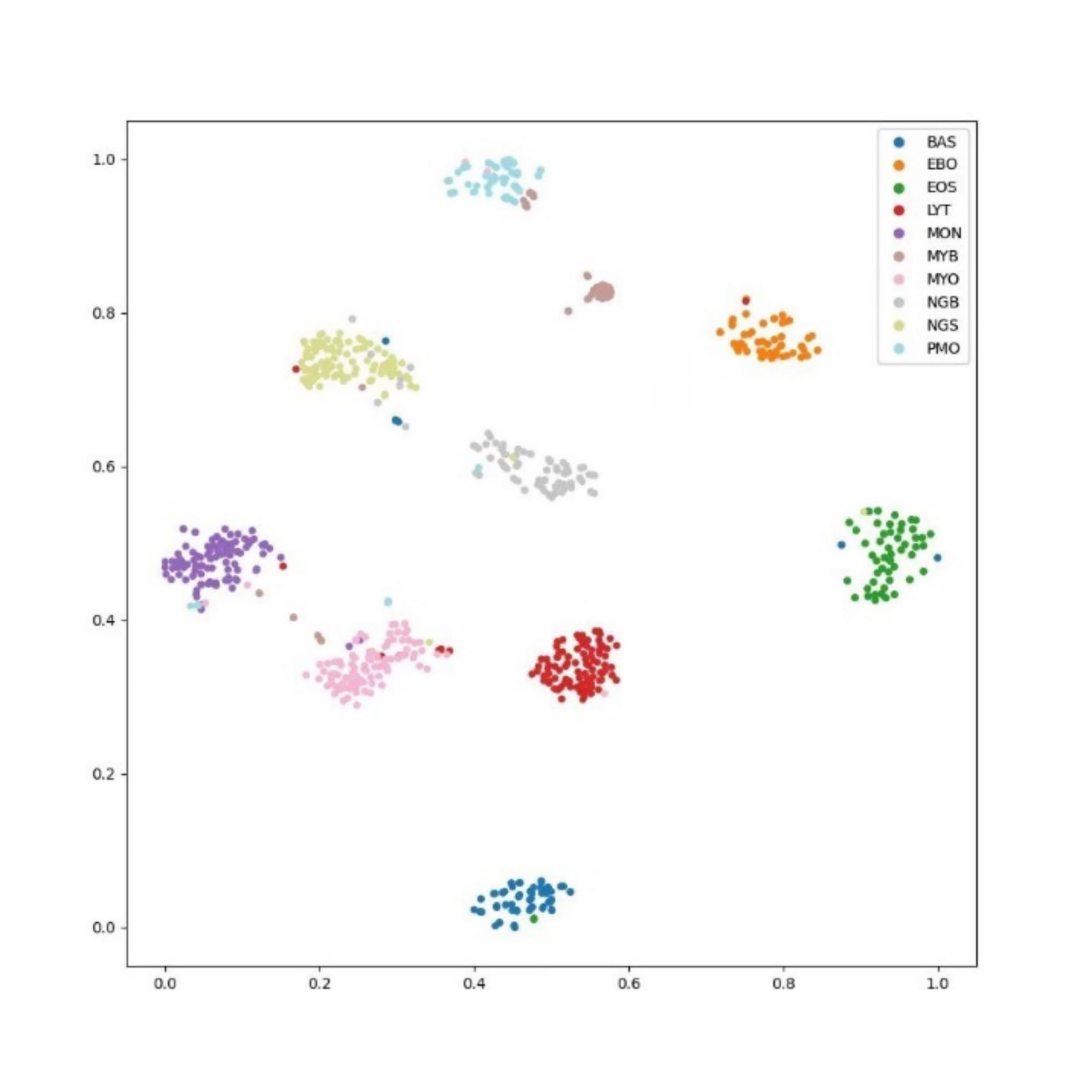
t-SNE dimensional reduction for categorical features with dynamic margins for subcenterArcFace Loss.

A comparison between the traditional softmax CE Loss and dynamic margins for subcenterArcface Loss is provided in Table 5. The implementation results indicate that the classification accuracy is improved by 0.59% using dynamic margins for subcenterArcface Loss. Figures 9 and 10 depict the t-SNE dimensionality reduction visualization results of the output classification features using softmax CE Loss and dynamic margins for subcenterArcface Loss, respectively. It can be observed that dynamic margins for subcenterArcface Loss achieve a more compact, class-to-class classification, facilitating better class separation. This loss function is thus more suitable for fine-grained image classification.

The choice of pre-training weights significantly affects the classification accuracy. As shown in Table 5, pre-training weights on the ensemble of TMAMD and RED4 using MAE yield better results compared to pre-training weights on Imagenet1K, resulting in a 0.52% improvement in classification accuracy.

## Discussion

This thesis validates the effectiveness of our proposed method for blood cell classification using two publicly available datasets. We compared several popular neural network models based on accuracy, precision, recall, F1-score, FLOPs, and parameters. As shown in Table 2, our model outperforms current mainstream models across all these metrics. Specifically, our model’s accuracy is 0.89 points higher than the second-best, precision is 1.8 points higher, recall is 0.89 points higher, and F1-score is 1.46 points higher.

Table 3; Fig. 7 indicate that our models for Erythroblast, Eosinophil, Lymphocyte, Monocyte, Myeloblast, and segmented neutrophil granulocytes exhibit excellent agreement with physician annotations, with precision and recall rates exceeding 90%. However, classifying granulocytes at different developmental stages remains challenging. For instance, Myelocytes are often misidentified as Promyelocytes or Myeloblasts due to subtle differences between these stages and the limited number of samples for Myelocytes and Promyelocytes. Basophils, similarly, are frequently misclassified as Eosinophils or segmented neutrophil granulocytes, likely due to the small number of Basophil samples.

While our method has demonstrated strong performance in cell classification, there are still limitations that warrant further research. The dataset we used is relatively small and limited in scope. Expanding the dataset with more diverse and larger single-cell samples could improve the model’s classification accuracy and robustness. Additionally, the effectiveness of MAE pre-training is highly dependent on data distribution and quality. Using datasets with more diverse distributions could lead to more generalized feature representations and thus better pre-training results.

In future research, we plan to explore two main areas. First, we will investigate the integration of state-space models, such as Mamba, with ViT or CNN to further improve classification accuracy. Second, we will collect datasets from a broader range of sources to investigate how our trained model can adapt to datasets with different distributions. This will involve a detailed exploration of domain adaptation within single-cell datasets that exhibit diverse distributions.

## Conclusion

This paper focuses on the fine-grained classification of blood cells, proposing an enhanced classification model based on Vision Transformer. The feature outputs of each layer of the Vision Transformer encoder are fused to enhance the model’s ability to represent fine-grained features. Additionally, the dynamic margins for subcenterArcface Loss function are employed to further improve the intra-class compactness and inter-class variability of the model. We also utilize the MAE self-supervised method to transfer pre-trained weights from two datasets, TMAMD and Red4, to our improved model. Our approach achieves state-of-the-art performance on both the TMAMD dataset and the Red4 dataset. Both qualitative and quantitative visualization results demonstrate the effectiveness and interpretability of our method. Compared to other recognition methods, our approach exhibits higher classification accuracy, providing a valuable reference for clinical diagnosis by doctors and holding potential clinical applications.

## Data Availability

The two public datasets used in this study are available in the following sites: (1)TMAMD: https://github.com/sty16/cell_transformer; (2) red4:https://github.com/wangye8899/BMEC; code availability: All codes of the proposed method are available at https://github.com/y070504/cells_cls.

## Declarations

**Correspondence** and requests for materials should be addressed to N.J. or Y.L.

### Reprints and permissions information

is available at www.nature.com/reprints.
